# Knowledge, attitudes and practices concerning breast cancer, cervical cancer and screening among healthcare professionals and students in Mogadishu, Somalia: a cross-sectional study

**DOI:** 10.3332/ecancer.2022.1455

**Published:** 2022-10-07

**Authors:** Lucas Walz, Deqo Mohamed, Adam Haibah, Nikhil Harle, Samir Al-Ali, Ayan Aden Moussa, Jude Alawa, Mohamed Abdullahi Awale, Kaveh Khoshnood

**Affiliations:** 1 Yale School of Public Health, Yale University, New Haven, CT 06510, USA; 2 Hagarla Institute, Mogadishu, Somalia; 3 Yale College, Yale University, New Haven, CT 06520, USA; 4 SIMAD University Faculty of Medicine and Health Sciences, SIMAD University, Mogadishu, Somalia; 5 Stanford University School of Medicine, Stanford, CA 94305, USA; * These authors contributed equally as first authors; † These authors contributed equally as senior authors

**Keywords:** breast cancer, cervical cancer, human papillomavirus, HPV vaccine, cancer, Somalia

## Abstract

**Introduction:**

Somali women face exceptionally high mortality and incidence rates from both breast cancer (BC) and cervical cancer (CC). They experience the highest age-standardised BC mortality rate in Africa and an age-standardised BC incidence rate of 41.7 per 100,000 women. Somalia’s second-highest cancer-related mortality and incidence rates are due to CC, both behind BC. It is critical to identify the underlying factors that may influence healthcare workers’ management of both cancers. At present, there is a lack of evidence regarding providers’ knowledge of these two cancers and their screening in Somalia.

**Methods:**

A cross-sectional questionnaire was administered with a purposive sampling strategy to 469 healthcare professionals and students and was completed by 405 (86%). Healthcare workers were recruited from Mogadishu-based hospitals.

**Results:**

One hundred and ninety-seven healthcare professionals and 207 students completed the survey and were included in the analysis. 89% and 73% of respondents demonstrated good knowledge of BC and CC, respectively. Only 46% knew that a vaccine could prevent CC, and 89% of healthcare professionals disagreed that human papillomavirus (HPV) vaccines were available to their patients. Attitudes towards cancer screening, in addition to breast self-examination (BSE), were overwhelmingly positive. For both BC and CC, 24% reported having treated a patient and 30% reported having conducted a screen for either disease.

**Conclusion:**

Overall, while knowledge of both diseases and screening was good, there remain areas for clear educational targeting such as HPV vaccine availability and BC preventability. Attitudes to screening for both diseases were exceedingly positive but, with the exception of BSE, failed to translate into practice due to inadequate resources and patient refusal. Future investments into Somalia’s chronic care management should prioritise technology necessary to conduct screenings for both diseases, expanding HPV vaccine access and understanding patients’ potential motivations for refusing screening.

## Introduction

Breast cancer (BC) mortality has increased rapidly in Africa, which experiences the highest age-standardised BC mortality rate of any continent [1]. In contrast to many high-income countries where the incidence and mortality of BC have remained fairly consistent, these measures have rapidly increased in lower-income countries whose populations are reaching older ages and adopting new lifestyle changes associated with BC including late child bearing and physical inactivity [2, 3]. Due to several contributing factors including scarce cancer diagnostic facilities [4], persistently long wait times [5] and limited cancer education [5, 6], many women present with late-stage disease, heightening their mortality risk.

Patients’ knowledge and attitudes toward BC are essential determinants of early detection, and it is well-established that the early diagnosis of BC is effective at reducing mortality rates [7, 8]. Yet, the evidence concerning their perceptions and knowledge of BC and screening programmes in some low- and middle-income countries (LMICs) remains sparse. Furthermore, breast self-examinations (BSEs) have been known as a cost-effective intervention with the potential to assist in the early detection of BC [9, 10], empower women to take a role in their health promotion [11, 12] and may encourage future professional breast examinations [13], but attitudes toward BSEs remain unexplored in many LMICs.

Similarly, cervical cancer (CC) is the leading cause of cancer-related deaths in women in many African countries [14], and the incidence of the disease has been found to be increasing in almost every sub-Saharan African country for which data are available [15]. The region is known to have the greatest age-standardised incidence rate of CC in the world, with about 90% of the world’s CC cases occurring in sub-Saharan Africa [15].

In part due to exceptionally high mortality and incidence rates they face from BC and CC, Somali women have some of the lowest health indicators in the world. They have a life expectancy of 59 years, compared to that of 64 for sub-Saharan African women overall [16]. They experience an age-standardised BC mortality rate of 29.1 per 100,000 women, the highest in all of Africa [17], and an age-standardised BC incidence rate of 41.7 per 100,000 women [1]. Somalia’s second-highest cancer-related mortality and incidence rates are due to CC, both behind BC [1]. Additionally, it is hypothesised that these numbers are underreported as qualitative studies of Somali refugees have demonstrated a preference of women to avoid CC screening due to a fear of the disease and a preference to die without knowing that they have cancer [18].

Somali women struggle to receive cancer treatment and diagnostic care from their country’s healthcare system due to a range of persistent barriers. Alongside a lack of consistent electricity at about half of surveyed healthcare facilities [19], unaffordable prices and a lack of regulation have resulted in general distrust towards the nation’s healthcare system and led to reports of poor care-seeking behaviour among Somalis [20]. Moreover, prolonged conflict by way of 30 years of civil war has severely damaged the country’s healthcare infrastructure, leaving its inhabitants vulnerable to climate-related disasters and a lack of consistent care for chronic conditions [21, 22]. Despite modelling studies having demonstrated the effectiveness of human papillomavirus (HPV) vaccines and widespread Pap testing in successful CC screening programmes [14, 15, 23], Somalia currently does not have an HPV vaccination programme and its Ministry of Health reports that HPV vaccines are unavailable throughout the country [24]. Additionally, informal conversations with hospital leaders in Mogadishu have revealed a widespread unavailability of CC screening equipment and a complete lack of functioning mammography equipment. This in turn forces physicians to utilise alternative BC screening methods such as clinical histories, or at select hospitals, ultrasounds and invasive biopsies which can prohibitively cost patients on the order of $100.

It is critical to explore the individual and institutional elements that may inhibit comprehensive cancer care in Somalia, especially as they pertain to healthcare workers’ knowledge and practice of early detection of both cancers. At present, assessments of providers’ knowledge of different women’s cancers and screening in the Horn of Africa provide conflicting reports. A study of Ethiopian female healthcare workers, for instance, demonstrated relatively high knowledge concerning CC [25], while another study among Eritrean nurses showed poor knowledge of BC risk factors [26]. A final study among urban health extension workers in central Ethiopia found that over half of respondents had BSE knowledge that did not necessarily translate into clinical practice [27].

To the authors’ knowledge, this study serves as the first investigating Somali healthcare professionals’ and students’ knowledge, attitudes and practices (KAP) towards BC, CC and screening in Somalia.

## Methods

### Design and instrument

A descriptive, cross-sectional KAP study among healthcare professionals and students concerning BC, CC and screening practices was carried out in Mogadishu, Somalia from 15 February 2021 until 28 March 2021. The 40-item survey tool consisted of eight sections: demographic information, KAP regarding both cancers and a separate section specific to BSE. Kress *et al* [28] examined the KAP concerning CC and screening among nurses, midwives, medical students, general practitioners, internists, paediatricians and obstetricians/gynaecologists in Ethiopian hospitals. Their survey was adapted to include questions pertaining to BC, BC screening and BSE from two other studies examining healthcare providers’ KAP in the African continent [26, 29].

In order to minimise experimenter bias, multilingual healthcare professionals from the Hagarla Institute, a Mogadishu-based non-profit organisation dedicated to furthering clinical research, capacity-building and skills transfer for medical personnel across Africa, underwent training as data collectors at SIMAD University’s Institute for Medical Research. The English questionnaire was available both in print and online format; this was translated to and from Somali language by the data collectors who were always present during the survey’s roughly 30-minute duration.

### Sample and setting

The survey was administered to 469 Mogadishu-based healthcare professionals and students. To counter bias stemming from nonresponse, an *a priori* determination was made to remove data from participants who did not respond to at least 85% of questions. This criterion was met by 405 participants, for a completion rate of 86%.

A purposive sampling method was utilised to survey healthcare professionals and students from healthcare-delivering institutions near Mogadishu, Somalia. Due to their direct involvement in Somali women’s potential cancer care, the survey aimed to receive responses from health professional students, midwives, general ward nurses, head nurses, obstetrician/gynaecologists, radiologists and general surgeons. The purposive sampling strategy was employed to compensate for an inability to access employee registries at participating institutions from which to randomly sample, and to ensure a sufficient amount of responses from a variety of patient-facing healthcare workers that provide BC or CC care. Participants were mostly recruited from four public and six private hospitals in Mogadishu.

### Data analysis

Responses were either collected directly in Qualtrics or were manually input onto the survey software in English. Data were exported for analysis on SAS Studio v.9.4 [30]. Categorical variables are reported using frequency and percentages of responses. To permit comparison with published investigations in neighbouring countries, a threshold of ≥50% of questions answered correctly was adopted to signify a ‘good’ knowledge level. An alpha of 0.05 was utilised for all analyses.

## Results

The survey received 405 complete responses. Participants who did not respond to at least 85% of the questions were removed from the analysis. All non-demographic questions had an option of ‘I don’t know’ or ‘prefer not to say’ to minimise this. One survey response from a participant who did not identify themselves as either a healthcare professional or student was excluded. The final analysis was conducted with 404 responses.

Of the included responses, 197 (49%) were self-identified healthcare professionals, and 207 (51%) were health professional students. Among healthcare professionals, the highest sampled professions were general ward nurses (25%) and midwives (19%). Many students (42%) did not associate with any of the provided options, likely due to participating in the study before differentiating into specialities. The mean age of participants was 29.9 (standard deviation ± 8.37), and 59% of respondents were female. Of practicing healthcare professionals, over 60% had been practicing for less than 5 years (Table 1). The most common affiliation among participants was with Banadir Hospital in Mogadishu.

Eighty-nine percent of included participants had a good knowledge of BC, correctly answering at least 9/18 questions pertaining to BC risk factors, severity and burden, while 61% showed superior knowledge by answering at least 12/18 questions correctly. Five questions were answered correctly by less than 50% of the sample. Two were regarding risk factors: First birth occurring after mother is ≥30 years old (49%); Use of oral contraceptive pills (46%), while the others related to those-at-risk and preventability of the disease: BC is preventable (8%); BC is most common among women in their 20s (45%); Only women can get BC (46%) (Table 2). Healthcare professionals and students differed in their knowledge of risk factors; professionals were significantly more likely to recognise five (non-) risk factors: History of smoking (92% versus 82%); Family history of BC (97% versus 89%); No history of breastfeeding (82% versus 73%); Having multiple sex partners (non-risk factor; 80% versus 65%); Larger breasts (non-risk factor; 65% versus 49%). Students correctly identified two risk factors at a significantly higher rate than healthcare professionals: First birth occurring after mother is ≥30 years old (36% versus 63%); Use of oral contraceptive pills (30% versus 63%).

Respondents had relatively less knowledge of CC. Seventy-three percent of respondents correctly answered at least 10/19 questions pertaining to CC, and 56% correctly responded to at least 12/19 questions. In addition to this poor overall performance, responses to CC questions exhibited more heterogeneity; more questions had significantly different response patterns across professional and student groups when compared to BC (Table 3). Healthcare professionals were more likely to correctly answer CC risk factor questions across the board but were less likely to correctly answer the following questions about CC screening and presentation: CC can usually be found at an early stage because of the obvious symptoms (false; 23% versus 33%); CC is most common among women in their 20s (false; 43% versus 62%). Professionals with more than 5 years of practice were more likely to show good knowledge of CC than their newer professional counterparts (100% versus 93%; *p* = 0.02). Also of note, a small majority of respondents correctly identified that there is a licensed vaccine that can prevent CC; healthcare professionals were significantly more likely to identify the existence of such a vaccine (65% versus 42%). Additionally, only 26% and 43% of respondents correctly identified that poor personal hygiene and use of intrauterine devices were not risk factors for CC, respectively.

Attitudes towards screening were overwhelmingly positive for both cancers. Over 98% of respondents agreed that both BC and CC are serious diseases, that screening for both diseases is an essential part of women’s healthcare, and that a screening programme for both cancers should be initiated in their community (Table 4). Relative to patients’ other needs, however, respondents were not as concerned about BC and CC screening, with over 92% stating that their patients have more pressing concerns than cancer screenings. Respondents also agreed that a lack of resources inhibited screening, with over 96% reporting that they lacked two or more fiscal or technological resources required for screening. Among other barriers to screening, 30% of respondents reported patients refusing opportunities for BC or CC screens.

**Figure 1.**
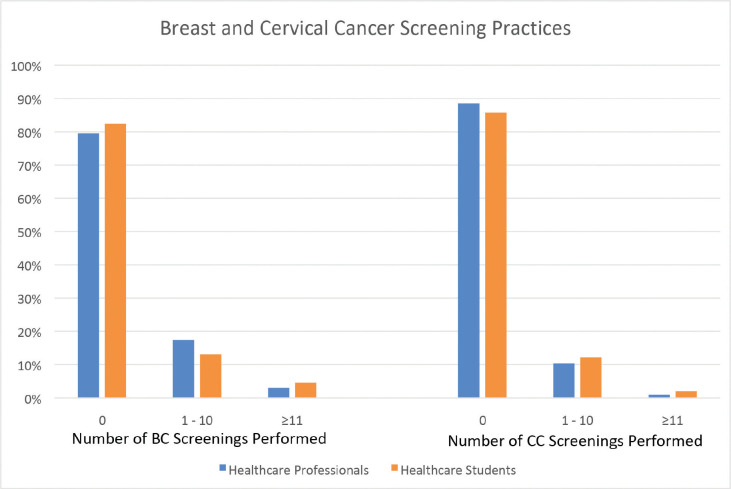
Proportion of medical doctors, nurses and midwives having performed BC and CC screening procedures. CC screening procedures included pap smear, HPV DNA testing, liquid-based cytology, visual inspection with acetic acid and visual inspection with Lugol’s solution.

Most respondents reported not having cared for BC and CC and precancerous patients. About 24% reported having treated either BC or CC patients (Table 5), while 30% reported having ever conducted either a BC or CC screening examination (Figure 1). Healthcare professionals with more than 5 years of experience were more likely than other professionals to have conducted a CC screening procedure (32% versus 17%; *p* = 0.02), and although they more frequently performed BC screenings (27% versus 16%), this difference was not significant. Moreover, over 95% of respondents had a positive attitude towards the efficacy of BSEs (Table 6); 92% were confident that they could detect abnormalities in breasts, and about 79% recommend that their patients conduct breast-examinations and felt confident about their ability to teach the technique to patients.

## Discussion

To the authors’ knowledge, this is the first study to investigate Somali healthcare professionals’ and students’ KAP towards BC, CC and screening for both diseases. A large majority (89%) of respondents demonstrated a good knowledge of BC prevention and screening, risk factors, severity and burden. This study provides evidence of Somali healthcare workers’ higher levels of BC knowledge compared to participants in nearby Northwest Ethiopia [34], Addis Ababa in Central Ethiopia [27, 35] and Eritrea [26]. As expected, healthcare professionals performed better than students at identifying BC risk factors. Surprisingly, a majority of healthcare professionals said that BC was most common in women in their 20s, potentially a reflection of growing trends of early-onset BC plaguing sub-Saharan African populations [36], and overwhelming majorities of both professionals (94%) and students (89%) stated that BC was a preventable disease. With regard to CC, a majority (73%) of healthcare professionals and students demonstrated good knowledge. Though lower than the BC scores, the current finding is higher than that of other sub-Saharan investigations in Tanzania and Côte d’Ivoire [37, 38], but lower than that of Ethiopia and Nigeria [25, 39, 40]. While only 54% of professionals and students knew that there was a vaccine available that could prevent CC, this is a higher rate than that identified in southern Ethiopia (36%) [25].

While Mogadishu-based medical universities’ curricula inform students about CC, BC, screenings and HPV, the widespread unavailability of screening equipment can make it difficult to reinforce these concepts with practice. It is therefore essential that medical education programmes all across Somalia incorporate lessons about risk factors and screening, as well as how to navigate sensitive conversations about women’s cancers with patients. In addition, the Somali Medical Association encourages attendance of biannual *Continuing Medical Education* events to reinforce established and introduce new concepts to graduated medical professionals [41]. These events may prove to be useful sites to incorporate lessons that help dispel misconceptions held by healthcare professionals, such as those identified in the present study surrounding BC preventability, CC risk factors and HPV vaccinations. Lamentably, the *Continuing Medical Education* events are only held in Somalia’s largest cities, and together with even greater unavailability of screening resources in rural areas and smaller cities, this gives reason to believe that healthcare professionals from other Somali provinces would demonstrate worse knowledge of both cancers and screening practices.

Attitudes towards screening of both diseases and of BSE proved remarkably positive. Though 99% and 98% of respondents stated that screening for BC and CC was an essential part of women’s healthcare, respectively, a lack of access to services necessary for prevention and early detection of both diseases was reported. Despite demonstrating this robust approval of screening, almost 70% of healthcare professionals and students disclosed having never conducted a screening for either disease, in part because over 97% reported a lack of access to essential screening services including laboratory equipment, money dedicated for screening tests and follow-up capacity. (A healthcare professional’s supervisory presence is required for Somali healthcare students to conduct screenings or offer recommendations; students exclusively assist professionals as part of a cancer patient’s treatment team.) An exception to this lack of screening practice was that of BSE, where 79% and 82% of respondents, respectively, reported frequently recommending that patients conduct BSEs and expressed confidence in their ability to teach the method. Without a comprehensive BC screening programme in place, widespread BSE may serve as an acceptable alternative for Somali women to become familiar with any changes in their breast, despite the method’s limitations.

While the availability of resources, both technological and human, reportedly played a substantial role in precluding healthcare workers from conducting screening, 30% of respondents reported a patient having refused screening for either cancer. These respondents concurred that patients refused screening for either cancer because it was looked down upon by friends or family, because they believed it would give them cancer and because they found it embarrassing, intrusive or painful, among other reasons. Studies aimed at evaluating BC and CC screening in the Somali context will therefore be essential – qualitative investigations aimed at uncovering the cultural nature of the stigma that inhibits screening for both cancers will be vital to effectively launch educational campaigns that dispel patients’ misconceptions to improve uptake of cancer screenings as their availability grows.

A monetary and technological investment is necessary to improve Somali healthcare workers’ ability to combat BC and CC – a needs assessment evaluating the current status and deficits of cancer screening infrastructure is indispensable for understanding where to direct new commitments from governmental and international institutions. Such an assessment could also help direct a dedication to comprehensive national HPV surveillance in Somalia, as data concerning HPV’s prevalence in at-risk populations or confirmed CC cases remain unavailable. Establishing robust HPV surveillance could incite organisations like GAVI, for which Somalia is eligible [42], to deliver aid in the form of HPV vaccinations and enhance primary and secondary CC prevention strategies.

This study was subject to several limitations. As the conducted investigation was cross-sectional and survey-based in nature, recall and social desirability biases are among the study’s potential limitations. The study utilised a purposive sampling technique to avoid neglecting any type of healthcare worker providing cancer care to Somali women, particularly midwives who play an invaluable role in providing reproductive health services across the nation [43]. It is not intended to be a representative sample of Somali healthcare workers and its generalisability is therefore limited. Lastly, a wave of COVID-19 cases plagued Somalia and the Mogadishu area in February and March of 2021, when the study was in the data collection stage; the added time and emotional strain may have influenced the selection of participants. We attempted to mitigate this by eliminating individuals who completed less than 85% of the survey, who may have been stressed for time and skipped past questions.

## Conclusions

This study is the first of its kind to examine the KAP of Somali healthcare workers as it relates to BC, CC and screening. While knowledge of both diseases and screening was generally good, there remain areas for clear educational targeting to improve the quality of chronic care provided by healthcare professionals and students, such as the preventability of BC and the existence of a vaccine that can prevent CC. Attitudes toward screening for both diseases were all exceedingly positive, but failed to translate into practice due to inadequate resources and patient refusal. Attitudes toward BSE were also remarkably positive and did translate to clinical recommendations. To expand access and increase the affordability of Somalia’s chronic care management, future investments should prioritise the technology necessary to conduct screenings for both diseases, expanding access to HPV vaccines and establishing an HPV surveillance programme and understanding patients’ potential motivations for refusing screening at the current moment.

## List of abbreviations

BC, Breast cancer; LMICs, Low- and middle-income countries; BSE, Breast self-examinations; CC, Cervical cancer; HPV, Human papillomavirus; KAP, Knowledge, attitudes and practices

## Conflicts of interest

The authors declare that they have no conflicts of interests. This article is based on a thesis project for completion of graduation requirements for a Master’s in Public Health.

## Funding

This was an unfunded study.

## Authors’ contributions

LW contributed to conceptualisation, formal analysis, methodology, software, supervision, validation, visualisation and writing. DM contributed to conceptualisation, investigation, project administration, resources, supervision and writing. AH contributed to data curation, investigation, project administration, resources and supervision. NK and SA contributed to conceptualisation, formal analysis, software, visualisation and writing. AAM contributed to data curation, project administration, resources and writing. JA contributed to conceptualisation, validation, visualisation and writing. MAA contributed to conceptualisation, investigation, methodology, project administration, resources, supervision, validation and writing. KK contributed to conceptualisation, formal analysis, methodology, project administration, resources, supervision, validation and writing.

## Ethics approval and consent to participate

This study received approval from the ethics board at SIMAD University’s Institute for Medical Research and was deemed exempt from review by the Yale IRB (ID #2000029594). Verbal informed consent was obtained by the researchers by way of a brief presentation regarding the investigation’s purpose, procedures and requirements for participation. Potential participants were made aware that participation was voluntary and could be withdrawn at any moment, that their responses would be anonymous and confidential, and that their participation would in no way impact their relationship with their employer. Data collectors received monetary compensation for their time and effort.

## Availability of data and materials

All data generated or analysed during this study are included in this published article and its supplementary information file (Supplementary Information: https://doi.org/10.6084/m9.figshare.20443374).

## Figures and Tables

**Table 1.** Participant demographics^a^.

Characteristic	Healthcare professionals (*n* = 197)	Healthcare students (*n* = 207)	Total (*n* = 404)
Age (years)			
≤25	27 (13.7)	118 (58.7)	145 (36.4)
26–35	106 (53.8)	67 (33.3)	173 (43.5)
36–45	50 (25.4)	11 (5.5)	61 (15.3)
≥46	14 (7.1)	5 (2.5)	19 (4.8)
Sex			
Male	85 (43.2)	81 (39.3)	166 (41.2)
Female	112 (56.8)	125 (60.7)	237 (58.8)
Profession			
Midwife	37 (18.8)	17 (8.2)	54 (13.4)
General ward nurse	50 (25.4)	45 (21.8)	95 (23.6)
Head nurse	23 (11.7)	12 (5.8)	35 (8.7)
General surgeon	17 (8.6)	20 (9.7)	37 (9.2)
Radiologist	26 (13.2)	5 (2.4)	31 (7.7)
Obstetrician/gynaecologist	23 (11.7)	21 (10.2)	44 (10.9)
Other	21 (10.7)	86 (41.8)	107 (26.6)
Licensed (certificate)^b^			
Yes	98 (50.0)	-	98 (50.0)
No	98 (50.0)	-	98 (50.0)
Years of practice**^b^**			
<1	25 (12.8)	-	25 (12.8)
1–5	94 (48.2)	-	94 (48.2)
6–10	52 (26.7)	-	52 (26.7)
≥11	24 (12.3)	-	24 (12.3)

a Values are frequency (column percent). Values may not add to 100 due to rounding

b Questions were only posed to healthcare professionals (as opposed to students)

**Table 2.** Knowledge concerning BC risk factors, aetiology and prevention.

Question	Healthcare professionals	Healthcare students	Total	*p*-value^b^
	**Correct answer^a^**			
History of smoking (risk factor)	176 (91.7)	154 (81.5)	330 (86.6)	0.0035
Family history of BC (risk factor)	191 (97.4)	172 (89.1)	363 (93.3)	0.0010
No history of breastfeeding (risk factor)	58 (81.9)	136 (73.1)	294 (77.6)	0.0413
Having multiple sex partners (non-risk factor)	144 (80.4)	112 (64.7)	256 (72.7)	0.0009
First birth occurring after mother is ≥30 years old (risk factor)	65 (36.1)	108 (63.2)	173 (49.3)	<0.0001
Obesity (risk factor)	137 (74.9)	122 (69.7)	259 (72.4)	n.s.
Increased age (risk factor)	134 (70.9)	125 (70.2)	259 (70.6)	n.s.
Use of oral contraceptive pills (risk factor)	56 (30.3)	108 (63.2)	164 (46.1)	<0.0001
Larger breasts (non-risk factor)	120 (64.9)	86 (48.6)	206 (56.9)	0.0018
BC is one of the leading causes of death in women worldwide (true)	187 (95.4)	173 (86.5)	360 (90.9)	0.0020
BC is preventable (false)	12 (6.2)	20 (10.9)	32 (8.5)	n.s.
It is possible to detect pre-cancerous breast tissue cells (true)	186 (95.4)	177 (92.1)	363 (93.8)	n.s.
The purpose of screening for BC is to detect pre-cancerous changes and the appearance of cancer (true)	180 (92.8)	174 (90.6)	354 (91.7)	n.s.
If untreated, BC can be fatal (true)	181 (93.8)	180 (93.3)	361 (93.5)	n.s.
BC is not curable (false)	143 (75.3)	130 (67.7)	273 (71.5)	n.s.
BC is most common among women in their 20s (false)	70 (37.8)	93 (52.8)	163 (45.2)	0.0042
Only women can get BC (false)	94 (49.0)	81 (43.6)	175 (46.3)	n.s.
Any woman is at risk for BC (true)	139 (72.4)	143 (77.7)	282 (75.0)	n.s.

a Values are frequency (percent). ‘I don’t know’ responses were coded as missing

b *p*-value is for *χ^2^* test between healthcare professionals and healthcare students

Source: [26, 29, 31–33]

**Table 3.** Knowledge concerning CC risk factors, aetiology and prevention.

Question	Healthcare professionals	Healthcare students	Total	*p*-value^b^
	**Correct answer^a^**			
Smoking cigarettes (risk factor)	175 (92.6)	128 (67.0)	303 (79.7)	<0.0001
Poor personal hygiene (non-risk factor)	48 (25.8)	50 (27.0)	98 (26.4)	n.s.
Having multiple sex partners (risk factor)	163 (86.7)	144 (80.0)	307 (83.4)	n.s.
Use of herbal remedies (non-risk factor)	111 (62.0)	74 (46.0)	185 (54.4)	0.0030
Use of tampons (non-risk factor)	141 (77.0)	107 (62.9)	248 (70.2)	0.0038
Infection with HPV (risk factor)	157 (82.2)	129 (77.2)	286 (79.9)	n.s.
Infection with HIV (risk factor)	142 (77.6)	105 (58.3)	247 (68.0)	<0.0001
Use of intrauterine devices (non-risk factor)	87 (48.3)	63 (37.3)	150 (43.0)	0.0371
CC is one of the leading causes of death in women worldwide (true)	161 (82.6)	140 (71.8)	301 (77.2)	0.0113
CC is preventable (true)	157 (83.1)	150 (83.8)	307 (83.4)	n.s.
It is possible to detect pre-cancerous cervical cells (true)	188 (96.4)	177 (94.2)	365 (95.3)	n.s.
The purpose of screening for CC is to detect pre-cancerous changes and the appearance of cancer (true)	183 (94.8)	178 (94.7)	361 (94.8)	n.s.
If untreated, CC can be fatal (true)	180 (93.3)	172 (90.5)	352 (91.9)	n.s.
CC is caused by a virus that is spread sexually (true)	136 (76.8)	100 (58.8)	236 (68.0)	0.0003
There is a vaccine that can prevent CC (true)	114 (65.1)	61 (41.5)	175 (54.4)	<0.0001
CC is not curable (false)	136 (70.1)	117 (65.7)	253 (68.0)	n.s.
CC is most common among women in their 20s (false)	81 (43.6)	112 (62.2)	193 (52.7)	0.0003
For CC, the progression of pre-cancerous cells to cancer can take 10–20 years (true)	73 (42.9)	75 (46.8)	148 (47.6)	n.s.
CC can usually be found at an early stage because of the obvious symptoms (false)	45 (23.4)	60 (33.0)	105 (28.1)	0.0404

a Values are frequency (percent). ‘I don’t know’ responses were coded as missing

b *p*-value is for *χ^2^* test between healthcare professionals and healthcare students

Source: [28]

**Table 4.** Attitudes concerning BC, CC and barriers to screening.

Question	Healthcare professionals	Healthcare students	Total	*p*-value^b^
	**Strongly/somewhat agree^a^**			
**Breast Cancer**				
BC screening is an essential part of women’s healthcare	193 (99.0)	201 (99.0)	394 (99.0)	n.s.
BC is a very serious disease	193 (99.0)	196 (99.5)	389 (99.2)	n.s.
A BC screening programme should be started or expanded in my community	193 (99.0)	190 (97.9)	383 (98.5)	n.s.
My patients have more pressing health problems than screening	187 (96.9)	166 (87.8)	353 (92.4)	0.0008
I have not had the necessary training in order to screen	138 (71.1)	156 (82.5)	294 (76.8)	0.0082
The screening tests are too expensive for my patients	126 (65.0)	148 (81.3)	274 (72.9)	0.0004
I do not have time or am too busy to screen	89 (45.9)	95 (51.4)	184 (48.6)	n.s.
I do not have the necessary equipment or supplies to screen	168 (86.2)	153 (79.3)	321 (82.7)	n.s.
The screening procedures are too difficult	96 (49.2)	100 (57.1)	196 (53.0)	n.s.
I do not have the necessary laboratory resources to screen	177 (92.2)	156 (84.3)	333 (88.3)	0.0174
I do not have the capacity to follow-up with patients after screening	116 (61.0)	135 (73.8)	251 (67.3)	0.0089
**Cervical Cancer**				
CC screening is an essential part of women’s healthcare	190 (100.0)	190 (96.0)	380 (97.9)	0.0051
CC is a very serious disease	191 (100.0)	188 (97.4)	379 (98.7)	0.0252
A CC screening programme should be started or expanded in my community	188 (99.5)	186 (97.4)	374 (98.4)	n.s.
My patients have more pressing health problems than screening	189 (97.4)	165 (88.2)	354 (92.9)	0.0005
I have not had the necessary training in order to screen	136 (69.7)	158 (81.4)	294 (75.6)	0.0072
The screening tests are too expensive for my patients	115 (59.9)	143 (77.3)	258 (68.4)	0.0003
I do not have time or am too busy to screen	78 (41.5)	86 (46.5)	164 (44.0)	n.s.
I do not have the necessary equipment or supplies to screen	172 (87.8)	158 (84.0)	330 (85.9)	n.s.
The screening procedures are too difficult	84 (43.8)	108 (61.4)	192 (52.2)	0.0007
I do not have the necessary laboratory resources to screen	174 (90.2)	148 (80.4)	322 (85.4)	0.0075
I do not have the capacity to follow-up with patients after screening	103 (54.8)	126 (67.7)	229 (61.2)	0.0101

a Values are frequency (percent). ‘I don’t know’ responses coded as missing

b *p*-value is for *χ^2^* test between healthcare professionals and healthcare students.

**Table 5.** Practices concerning BC and CC diagnosis and treatment^c^.

Question	Healthcare professionals	Healthcare students	Total	*p*-value^b^
	**Yes^a^**			
**Breast Cancer**				
Have you ever diagnosed a patient with pre-cancerous breast lesions?	13 (11.2)	20 (12.7)	33 (12.0)	n.s.
Have you ever treated a patient with pre-cancerous breast lesions?	9 (7.8)	14 (9.2)	23 (8.6)	n.s.
Have you ever diagnosed a patient with BC?	16 (13.8)	21 (13.7)	37 (13.8)	n.s.
Have you ever treated a patient with BC?	17 (14.5)	16 (10.8)	33 (12.4)	n.s.
**Cervical Cancer**				
Have you ever diagnosed a patient with pre-cancerous cervical lesions?	29 (14.7)	37 (18.3)	66 (16.5)	n.s.
Have you ever treated a patient with pre-cancerous cervical lesions?	29 (14.7)	32 (16.2)	61 (15.5)	n.s.
Have you ever diagnosed a patient with CC?	43 (21.9)	33 (16.8)	76 (19.4)	n.s.
Have you ever treated a patient with CC?	42 (21.5)	28 (14.5)	70 (18.0)	n.s.

a Values are frequency (percent). ‘I don’t know’ responses were coded as missing

b *p*-value is for *χ^2^* test between healthcare professionals and healthcare students

c A healthcare professional’s supervisory presence is required for Somali healthcare students to conduct screenings or offer recommendations. Students exclusively assist professionals as part of a cancer patient’s treatment team.

**Table 6.** Attitudes and practices towards BSE^c^.

Question	Healthcare professionals	Healthcare students	Total	*p*-value^b^
	**Strongly/somewhat agree^a^**			
Have you heard of BSEs? (Yes^d^)	164 (85.0)	152 (78.8)	316 (81.9)	n.s.
BSE is a useful tool for the early detection of BC	183 (97.3)	183 (94.3)	366 (95.8)	n.s.
BSE is difficult and time consuming	43 (24.4)	70 (38.0)	113 (31.4)	0.0054
I am confident that I could detect abnormalities in a breast if there were any	178 (97.3)	158 (87.3)	336 (92.3)	0.0004
I am confident I could teach someone to conduct their own BSE	150 (81.1)	160 (83.3)	310 (82.2)	n.s.
I frequently recommend that patient conduct their own BSE	142 (82.6)	139 (75.1)	281 (78.7)	n.s.

a Values are frequency (percent). ‘I don’t know’ responses were coded as missing

b *p*-value is for *χ^2^* test between healthcare professionals and healthcare students

c A healthcare professional’s supervisory presence is required for Somali healthcare students to conduct screenings, provide treatment or offer recommendations

d Answers shown in table for question 1 correspond to the response: ‘Yes’
